# Successful resolution of refractory chronic cough induced by gastroesophageal reflux with treatment of baclofen

**DOI:** 10.1186/1745-9974-8-8

**Published:** 2012-10-18

**Authors:** Xianghuai Xu, Qiang Chen, Siwei Liang, Hanjing LÜ, Zhongmin Qiu

**Affiliations:** 1 Department of Respiratory Medicine, Tongji Hospital, Tongji University School of Medicine, No. 389 Xincun Road, Shanghai, 200065, China

**Keywords:** Baclofen, Chronic cough, Gastroesophageal reflux, Multi-channel intraluminal impedance combined with pH monitoring

## Abstract

Gastroesophageal reflux induced cough is a common cause of chronic cough, and proton pump inhibitors are a standard therapy. However, the patients unresponsive to the standard therapy are difficult to treat and remain a challenge to doctors. Here, we summarized the experience of successful resolution of refractory chronic cough due to gastroesophageal reflux with baclofen in three patients. It is concluded that baclofen may be a viable option for gastroesophageal reflux induced cough unresponsive to proton pump inhibitor therapy.

## Introduction

Gastroesophageal reflux induced cough (GERC) is a common cause of chronic cough and accounts for 5-41% of chronic cough. [1] Although proton pump inhibitors are a standard therapy for GERC, some patients do not respond to the antireflux medical treatment and remain a challenge to doctors. Here, we report three patients with GERC resistant to proton pump inhibitors but successfully treated with baclofen.

## Case presentations

### Patient 1

A 26-year-old male patient with persistent cough for 3.5 years was referred to our respiratory clinic. He complained of cough day and night, with small amounts of viscous sputum. The accompanying symptoms included frequent clearing of the throat, occasional heartburn and acid regurgitation, but no post-nasal drip and chest pain (Table 1). The patient acknowledged a medical history of allergic rhinitis for 7 years, denied any exposure to environmental or occupational irritants, and never smoked. One year prior to the visit, he received a full diagnostic work-up in another respiratory clinic, where the results of laboratory investigations showed the normal chest radiographs and lung function, negative bronchial provocation to methacholine, positive IgE specific to dust mite and soybean in the serum, 1.5% of eosinophils in induced sputum, normal findings in fiberobronchoscope, and only submucosal edema with infiltration of scattered eosinophils and lymphocytes into airway mucosa as described by the pathologist examining the biopsy examples. A further 24-hour esophageal pH monitoring revealed an abnormal acid-reflux with a DeMeester score of 61.4 and the symptom association probability of 99%. Then, the presumptive diagnosis of GERC was established and oral omeprazole 20 mg twice a day was commenced. Three months later, his heartburn and acid regurgitation disappeared while the cough did not improve. The patient’s cough persisted despite the subsequent treatment with oral montelukast and inhaled corticosteroid.

**Table 1:** Clinical characteristics of the patients

	**Patient 1**	**Patient 2**	**Patient 3**
Ages (yr)	26	63	42
Gender (M/F)	M	F	M
Cough duration (month)	42	24	25
Heartburn	Yes	Yes	No
Regurgitation	Yes	Yes	Yes
Cough symptom score			
Daytime	3	4	3
Nighttime	1	2	2
Cough threshold to capsaicin			
C2(μmol/L)	0.49	0.49	1.95
C5(μmol/L)	31.2	1.95	7.8
FEV_1_ (%predicted)	99.1	101.8	86.1
FVC (%predicted)	96.9	104.0	96.1
FEV_1_/FVC (%)	89.5	83.8	75.0
PD_20_FEV_1_(μmol)	>7.8	>7.8	>7.8

FEV1: forced expiratory volume in one second; FVC: forced vital capacity; PD_20_FEV_1_: the cumulative provocative dose of histamine causing a 20% fall in FEV_1_.

**Figure 1:**
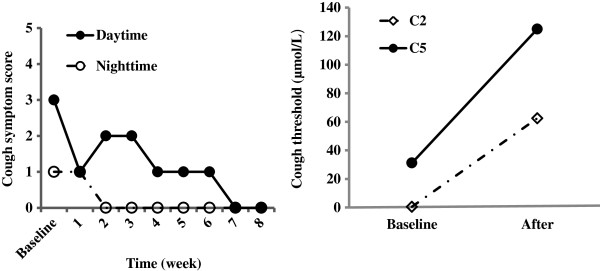
Changes in cough symptom score against the duration of the treatment with baclofen, and cough threshold C2 and C5 to capsaicin when cough was resolved in Patient 1.

Physical examination showed no other abnormal findings except for erythema and “cobblestone” appearance on the posterior pharyngeal mucosa. The repeated lung function testing and bronchial challenge with histamine were normal. Cytology in the induced sputum showed 41.5% of monocytes, 56.5% of lymphocytes and 2.0% of eosinophils. The patient received a 2 week course of oral chlorpheniramine, 4 mg three times a day for a possible upper airway cough syndrome and then one week of oral prednisone 25 mg daily for suspected eosinophilic bronchitis. Since no relief of cough was observed, multi-channel intraluminal impedance combined with pH monitoring (MII-pH) was ordered, and the abnormal non-acid reflux and positive symptom association probability for non-acid reflux was found (Table 2). After 8 weeks of additional augmented antireflux medical therapy comprising omeprazole, 20 mg twice a day and domperidone, 10 mg three times a day, the patient did not feel any improvement. Then, baclofen, 20 mg three times a day was used to replace domperidone. The patient's coughing decreased noticeably in a week, and completely resolved in two months, as indicated by the decreased cough symptom score and cough reflex sensitivity to capsaicin (Figure 1). Within the next four months of follow-up, no reoccurrence of cough was reported. The dosage of baclofen has been reduced to 20 mg daily. No discernable side effects were noted during the treatment with baclofen.

**Table 2:** The results of multi-channel intraluminal impedance combined with pH monitoring in the patients

	**Patient 1**	**Patient 2**	**Patient 3**	**Reference value**
DeMeester score	0.70	168.13	20.22	<14.72
SAP for acid reflux (%)	0.0	97.3	84.4	<95
SAP for nonacid reflux (%)	95.2	0.0	0.0	<95
Acidic reflux (n)	22.8	41.6	36.3	10-35
Weakly acidic reflux (n)	29.0	8.1	8.0	5-18
Weakly alkaline reflux (n)	23.4	2.3	1.1	1-7
Gas reflux (n)	26.2	13.9	11.4	3-17
Liquid reflux (n)	11.0	25.4	8.0	10-32
Mixed reflux (n)	64.6	26.6	37.5	11-26
Proximal extent (n)	3.0	5.2	4.3	4-17
Bolus exposure (%)	1.0	2.3	2.1	0.4-1.2
Bolus clearance (s)	10.0	9.0	14.5	8-13

SAP: symptom association probability.

### Patient 2

A 63-year-old housewife with a 2-year history of dry cough was referred for the suspicious GERC. She described that cough occurred through day and night, and disturbed her sleep, with the occasional heartburn, regurgitation and urinary incontinence (Table 1). The triggers included the cold and smoky air. A past medical history of hypertension and diabetes was present, but angiotensin-converting enzyme inhibitors were not currently being taken. On physical examination, only the follicular hyperplasia was found on the posterior pharyngeal mucosa. Her chest radiographs and lung function testing were normal, bronchial provocation with histamine did not demonstrate the existence of airway hyperresponsiveness. Cell differentials in the induced sputum consisted of 60.5% of monocytes, 37.0% of lymphocytes, 2.0% of neutrophils and 0.5% of eosinophils. MII-pH showed a serious abnormal acid reflux and positive symptom association probability for acid reflux (Table 2). When waiting for the results of the laboratory investigations, the patients was treated with antihistamine/ decongestant for a week, but without success.

**Figure 2:**
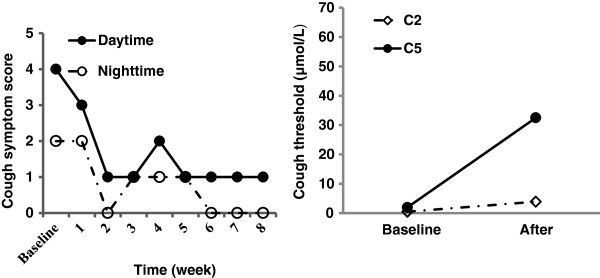
Changes in cough symptom score against the duration of the treatment with baclofen, and cough threshold C2 and C5 to capsaicin when cough was resolved in Patient 2.

No improvement in her cough and gastrointestinal symptoms was noted with 8 weeks course of initial antireflux medical treatment comprising omeprazole and domperidone. Baclofen, 20 mg three times a day, was given to substitute for domperidone. The patient`s cough attenuated significantly in two weeks, and resolved in three months, in companion with the improvement of cough symptom score and cough reflex sensitivity to capsaicin (Figure 2). At 4-month review after the initiation of baclofen, the patient only complained of the occasional mild cough. The dizziness was reported in the beginning of the treatment with baclofen, but it was tolerable and waned in the two weeks.

### Patient 3

A 42 year-old man with a 2-year history of persistent dry cough was referred for the diagnosis of cause. He coughed all the day, severely at postprandial time or when going to bed. An occasional regurgitation was described but without heartburn or the sensation of postnasal drip (Table 1). The patient was a lifetime nonsmoker, and had no history of chronic rhinitis or occupational exposure to pollutants. Six months ago, he visited to another respiratory clinic, where chest X-ray and lung function examinations were within the normal limits, and bronchial challenge confirmed airway hyperresponsiveness, as shown by 3.0 μmol of the cumulative provocative dose of histamine causing a 20% fall in FEV_1_. Then, the diagnosis of cough variant asthma was considered. However, the anti-asthma therapy including the initial inhalation of salmeterol/fluticasone 50/250μg, twice a day for one month and the subsequent trials with oral prednisone and montelukast failed to relieve the cough.

Physical examination was normal. The repeated bronchial provocation with histamine demonstrated the recovery of airway responsiveness. Cell examination in the induced sputum showed 82.5% of macrophages,12.0% of lymphocytes and 5.5% of neutrophils. MII-pH study was performed which revealed a slight abnormal acid reflux and the marginal level of symptom association probability (Table 2).

**Figure 3:**
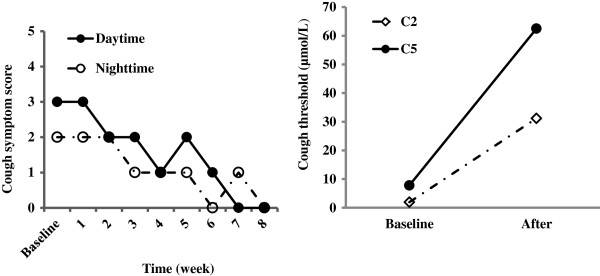
Changes in cough symptom score against the duration of the treatment with baclofen, and cough threshold C2 and C5 to capsaicin when cough was resolved in Patient 3.

The initial trial with omeprazole plus domperidone was unsuccessful. Thus, baclofen was added to the therapy while domperidone was withdrawn. Cough significantly improved within the 4 weeks and completely resolved within the two months. The effectiveness was also verified by the decreases in cough symptom score and cough reflex sensitivity to capsaicin (Figure 3). The dosage of baclofen was maintained for two months after the resolution of cough, and then began to reduce in a speed of 10mg a week. Since cough did not relapse with the minimum dose of 20 mg daily, baclofen was stopped two months later. No obvious adverse effect except for a slight sleepiness was reported during the treatment with baclofen.

## Discussion

In the patients with presumptive GERC whose cough persists despite proton pump inhibitors therapy, three possibilities arise to explain the continuing cough: 1. the gastric acid may be incompletely suppressed, and the patients are having continuing acid reflux that causes cough by microaspiration or esophageal-tracheobronchial reflex [1]. 2. non-acid (weakly acidic or alkaline) reflux is producing cough. It is proposed that cough due to non-acid reflux is resistant to proton pump inhibitors since they only reduce the pH of the refluxate but not the amount and the rate of reflux episodes [2]. 3. the ongoing cough is not related to any continuing reflux. It has been reported that only 40.8% of the patients with the positive findings of 24 hour esophageal pH monitoring were responsive to the high dose of omeprazole [3]. In all the three patients, MII-pH uncovered the abnormal acid or nonacid reflux and the temporal association between reflux and cough. Therefore, GERC could be established since the other common causes of chronic cough such as upper airway cough syndrome, cough variant asthma and eosinophilic bronchitis were excluded with the previous investigation procedure and specific therapies. The diagnosis of GERC was finally confirmed by the favorable response to the subsequent antireflux treatment containing omeprazole and baclofen even though the initial standard antiacid medical treatment failed.

In theory, the therapeutic options available for refractory GERC resistant to proton pump inhibitors include prokinetic agents, transient lower esophageal sphincter relaxation inhibitors and antireflux surgery because they all have the ability to reduce the frequency of reflux and volume of refluxate. At present, the efficacy of prokinetic agents has not been ascertained, which can explain the failure of the initial antireflux therapy containing domperidone. Antireflux surgery should not be the preferred choice when a cause and effect relationship between reflux and chronic cough is not definitely established. We selected baclofen, a potent inhibitor of transient lower esophageal sphincter relaxation, as an add-on trial for GERC unresponsive to proton pump inhibitors since it has been proposed in the management of difficult to treat gastroesophageal reflux disesase [4]. To our knowledge, this is the first report for the successful resolution of refractory GERC with baclofen.

Baclofen modulates the transient lower esophageal sphincter relaxations mediated by vagal reflex pathways through the activation of gamma-aminobutyric acid B receptor. Considering transient lower esophageal sphincter relaxations account for the vast majority of reflux events [5], it is predictable that baclofen may be helpful for refractory GERC by the inhibition of both acid and nonacid reflux. The limited studies have shown that it decreased the frequency of transient lower esophageal sphincter relaxations by 40-60% and acid or non-acid reflux episodes by 43% [6-8]. Vela has illustrated that baclofen reduced the symptoms related to acid reflux by 72% and related non-acid reflux by 21% in a small cohort of patients with heartburn [9]. In addition, baclofen has also been recognized for a long time to have a nonspecific antitussive activity both in animals [10] and in humans [11,12]. There have been several lines of evidence illustrating baclofen can treat the cough caused by angiotensin-converting enzyme inhibitors [13] and refractory chronic cough with unknown cause [14]. Our observations revealed that 2–4 week course of baclofen was needed for the significant alleviation of cough, and the complete resolution was generally achieved in the 2–3 months. Because MII-pH is an invasive procedure, our patients refused to undergo a repeated examination after the cough was resolved. Therefore, we were unable to directly evaluate the inhibitory efficacy of baclofen on acid or non-acid reflux in the patients. Nevertheless, it can be speculated that baclofen may play a therapeutic role through the effective blockade of all types of reflux events as well as the nonspecific antitussive effect.

A variety of central nervous system-related side effects can be produced by Baclofen, including somnolence, dizziness, fatigue, weakness and trembling. The other adverse reactions consist of dry mouth, nausea, vomiting, diarrhea or constipation. These side effects usually occur in the early or high-dose phase of treatment, and often limit the utility of baclofen in clinical practice [4]. In our patients, there was only slight dizziness and sleepiness in patient 2 and 3 respectively, which did not interrupt the treatment of baclofen. The lack of obvious side effects may be attributed to the well tolerance of the patients to the drug.

In conclusion, baclofen may be a viable option for refractory cough due to gastroesophageal reflux. Further study is needed to validate its therapeutic efficacy for GERC in the future.

## Consent

Written informed consent was obtained from the patient for publication of this report and any accompanying images.

## Competing interests

The authors declare that they have no competing interests.

## Authors' contributions

XX was in charge of collection of cases and writing the manuscript. QC, SL and HL took part in the collection of cases and review of the manuscript. ZQ was in charge of design and coordination of the program, review and correction of the manuscript. All authors read and approved the final manuscript.
